# Mannan-Binding Lectin Regulates Inflammatory Cytokine Production, Proliferation, and Cytotoxicity of Human Peripheral Natural Killer Cells

**DOI:** 10.1155/2019/6738286

**Published:** 2019-12-13

**Authors:** Jia Zhou, Mengyao Hu, Jie Li, Yan Liu, Jialiang Luo, Liyun Zhang, Xiao Lu, Daming Zuo, Zhengliang Chen

**Affiliations:** ^1^Department of Immunology, School of Basic Medical Sciences, Southern Medical University, Guangzhou, Guangdong 510515, China; ^2^The Third Affiliate Hospital of Southern Medical University (Academy of Orthopedics Guangdong Province), Guangzhou, Guangdong 510000, China; ^3^Instituteof Molecular Immunology, School of Laboratory Medicine and Biotechnology, Southern Medical University, Guangzhou, Guangdong 510515, China

## Abstract

Natural killer (NK) cells represent the founding members of innate lymphoid cells (ILC) and play critical roles in inflammation and the immune response. NK cell effector functions are regulated and fine-tuned by various immune modulators. Mannan (or mannose)-binding lectin (MBL), a soluble C-type lectin, is traditionally recognized as an initiator of the complement pathway. Recently, it is also considered as an immunomodulator by its interaction with kinds of immune cells. However, the effect of MBL on NK cell function remains unexplored. In this study, we found that human plasma MBL could interact directly with peripheral NK cells partially via its collagen-like region (CLR). This MBL binding markedly suppressed the interleukin-2- (IL-2-) induced inflammatory cytokine tumor necrosis factor-alpha (TNF-*α*) and interferon-gamma (IFN-*γ*) production but increased the IL-10 production in NK cells. In addition, the expression of activation surface markers such as CD25 and CD69 declined after MBL treatment. Also, MBL impaired the proliferation and lymphokine-activated killing (LAK) of NK cells. Moreover, we demonstrated that MBL inhibited IL-2-induced signal transducers and activators of transcription 5 (STAT5) activation in NK cells. In conclusion, we have uncovered a far unknown regulatory role of MBL on NK cells, a new clue that could be important in the immunomodulatory networks of immune responses.

## 1. Introduction

Natural killer (NK) cells belong to the family of innate lymphoid cells (ILC) and play an important arm of the immune system [1]. They can exert their cytotoxicity against the infected, transformed, or otherwise “stressed” cells without presensitization or MHC restriction [2]. Their constitutive storage of perforin and granzymes, as well as the rapid production of IFN-*γ* and TNF-*α* upon priming, allows their prompt intervention against target cells and the initiation of inflammation [3]. Moreover, they also exhibit their immunomodulatory function, affecting other cells and acting as a link between the adaptive and innate immunity [4]. Of note, they represent a promising target for immunotherapy since their critical role in defense of tumor and infection [5]. Nevertheless, they can also be detrimental to the host, contributing to the development of immune disorders [6]. Therefore, under pathological conditions and during inflammation, NK cells extravasate into the lymph nodes and accumulate at the inflammatory or tumor site, playing the complex yet critical physiological roles [7]. Importantly, the development, survival, proliferation, and effector functions of NK cells are critically dependent on cytokines, such as IL-2, IL-12, IL-15, and IL-18, secreted by other cells of the immune system [8]. NK cell activity is also controlled by the integration of signals arising from the activating and inhibitory receptors in the education process [9]. Moreover, resting NK cell functional priming is regulated by other various immune modulators in the immune system [10]. However, the mechanisms that control NK cell activity in the immune regulatory networks had not yet been fully understood.

Mannan (or mannose)-binding lectin (MBL), a prototypic pattern recognition molecule, can enhance phagocytosis of microorganisms by leukocytes and activate the lectin complement pathway [11]. MBL deficiency is a common complement deficiency in humans [12]. Increased susceptibility to infection, higher incidence, and worse prognosis of severe sepsis/septic shock appear to be associated with low-producer haplotypes of MBL [13]. Moreover, plasma MBL substitution restored the observed opsonic function defect in some MBL-deficient patients [14]. However, an excess of MBL levels/activation sometimes might be harmful due to the possibility of an unbalanced inflammatory response and the tissue injury [15]. Therefore, the functional role of MBL needs to be further investigated.

In addition, MBL can also modulate the host immune response independent of complement activation. Our previous studies demonstrated that MBL could bind to human monocyte and attenuate inflammatory response [16, 17]. Indeed, monocyte-derived DC from individuals with MBL deficiency showed an enhanced proinflammatory cytokine production in response to microbial stimulation [18]. Recently, we demonstrated that MBL ablation in mice could exacerbate sterile liver inflammation [19]. The other *in vivo* study also found that injection of recombinant MBL in mice could regulate the host immune response [20]. Also, our recent study revealed that MBL interaction with human T cells could suppress T cell activation [21]. Therefore, MBL represents a pleiotropic immunomodulator affecting numerous cell types of innate and adaptive immunity. However, whether and how MBL affect NK cell function has not yet been elucidated.

To address this issue, we explored the effect of MBL on NK cell activity. Here, we found that MBL could bind to NK cells partially via its CLR. Interestingly, this interaction attenuated the inflammatory cytokine induction and inhibited the NK cell proliferation, activation, and cytotoxicity induced by IL-2. And this NK cell activity impairment was accompanied by the decrease of STAT5 phosphorylation in NK cells. Collectively, our data showed that MBL engagement could regulate the NK cell activity and unraveled a new mechanism of MBL as a regulator of the immune response.

## 2. Materials and Methods

### 2.1. Preparation of MBL

MBL was isolated from human plasma by affinity chromatography on a mannan-agarose column (Sigma-Aldrich, St. Louis, MO, USA) and subsequent anion-exchange chromatography using MonoQ HR 5/5 column (Pharmacia Biotech Europe, Orsay, France) [21]. Human serum albumin (HSA) was prepared as we previously described [22]. Recombinant CRD or collagen-like region (CLR) of MBL was expressed in *E. coli* using the pET expression system (Novagen, Madison, WI, USA) and purified by nickel-chelating resins (GE Healthcare, Piscataway, NJ, USA) according to the manufacturers' protocols [17]. Possible residual endotoxin in the purified proteins was removed by the Detoxi-Gel Endotoxin Removing Column (Pierce, Rockford, IL, USA), and endotoxin level in the protein preparations was undetected by a Limulus Amebocyte Lysate kit (BioWhittaker, Walkersville, MD, USA). The functional activity of the purified MBL was determined by using an ELISA-based technique for the evaluation of mannan-binding capacity [23]. In brief, purified MBL were added to plates precoated with mannan and subsequently incubated with mouse anti-MBL antibody (clone 3B6, Abcam, Cambridge, MA, USA). The levels of bound MBL were determined using colorimetric assays after incubation with goat anti-mouse IgG H&L (HRP) (Abcam, Cambridge, MA, USA).

Bio-MBL or bio-HSA was prepared using EZ-Link Sulfo-NHS-LC-LC-Biotin following the manufacturer's instructions (Thermo Scientific, Rockford, USA). In brief, the biotin and protein mixture (80 *μ*g biotin/mg protein) was incubated on ice for two hours; then, the excess nonreacted and hydrolyzed biotin was removed by dialysis using PBS for 2 days.

### 2.2. Cell Preparation

Peripheral blood mononuclear cells (PBMCs) were isolated from whole blood samples collected from healthy donors by Ficoll density gradient centrifugation as we reported before [17]. The study was reviewed and approved by the Medical Ethics Committee of Southern Medical University. Before the collection of the blood sample, informed consent for taking part in the study was obtained from each participant. Purified NK cells were isolated from PBMCs using NK Cell Isolation Kit in the MACS column purification systems according to the manufacturer's instructions (Miltenyi Biotec, Bergisch Gladbach, Germany). Enriched cells were >95% pure. Purified NK cells were suspended in RPMI 1640 (Gibco BRL, Gaithersburg, MD, USA) supplemented with 2 mM L-glutamine, 100 U/ml of penicillin, 100 *μ*g/ml of streptomycin, and 10% heat-inactivated fetal bovine serum (FBS, Gibco, Grand Island, NY), then incubated in 96-well U-bottom plates (2 × 10^5^ cells/well). Human K562 cell line was obtained from the American Type Culture Collection and maintained in RPMI 1640 medium supplemented with 2 mM L-glutamine, 100 U/ml of penicillin, 100 *μ*g/ml of streptomycin, and 10% FBS.

### 2.3. Analysis of MBL Binding to NK Cells

For each sample, 2 × 10^5^ purified NK cells were resuspended in Tris-buffered saline (TBS, pH 7.4), supplemented with 1% bovine serum albumin (BSA) and subsequently incubated with indicated concentration of bio-MBL or bio-HSA at 4°C for 1 h, followed by washing with TBS to remove the unbound proteins. Cells were then stained with streptavidin-FITC (BD Biosciences Pharmingen, San Diego, CA). The binding of MBL was analyzed by FACSCalibur (Becton Dickinson, Mountain View, CA, USA); cells stained with streptavidin-FITC served as the control. For competition studies, NK cells were preincubated with 50 *μ*g/ml mannan, CLR, or CRD of MBL 4°C for 30 min.

For ELISA, 2 × 10^5^ purified NK cells were pretreated with or without CLR of MBL at 4°C for 30 min, followed by incubation with indicated concentrations of MBL at 4°C for 1 h, then washed with TBS to remove the unbound proteins. Subsequently, the cells were coated onto ELISA wells (Nunc, Kamstrup, Denmark) by centrifugation and subsequently fixed with 4% paraformaldehyde for 15 min at RT. After that, the cells were washed with TBS and then incubated with mouse anti-MBL antibody (3B6) following by goat anti-mouse IgG H&L (HRP). The levels of bound MBL were determined using colorimetric assays.

### 2.4. Cell Proliferation and Cytotoxicity Assays for LAK Activity

The NK cell proliferation level was evaluated using Cell Counting Kit-8 (CCK-8) (Dojindo, Tokyo, Japan) analysis, the 5-carboxy-fluorescein diacetate succinimidyl ester (CFSE, Molecular Probes, Eugene, OR, USA) dividing method, or Ki67 measurement. Briefly, purified NK cells were labeled with 1 *μ*M CFSE at 37°C for 8 min or left unlabeled, followed by treatment with the indicated concentration of MBL for 15 min at 37°C or not. Subsequently, the cells were incubated with 100 U/ml human recombinant IL-2 (PeproTech, Rocky Hill, NJ). Five days later, cells were harvested for Ki67 measurement by FACS or further cytotoxicity assays.

For cytotoxicity assays, CFSE-labeled NK (E) cells stimulated for 5 days as described above were cocultured with 2 × 10^5^ their natural target K562 cells (T) at the E/T ratio of 5 : 1, followed by staining with 7-aminoactinomycin D (7-AAD, BD Biosciences Pharmingen, San Diego, CA) and analyzed by FACS. Cytotoxicity was calculated as %specific lysis = [(%sample lysis–%basal lysis) × (100 − %basal lysis)].

### 2.5. Flow Cytometric Analysis

Fluorochrome-conjugated mouse anti-human monoclonal antibodies (mAbs) were used: anti-CD3-APC (clone UCHT1), anti-CD25-FITC (clone MA-251), anti-CD56-PE (clone B159), anti-CD69-APC (clone FN50), anti-NKp30-PE (clone p30-15), anti-NKp46-PE (clone 9-E2) or relevant control mAbs (BD Pharmingen, San Diego, CA), anti-CD56-FITC (clone BC96), anti-CD3-PE-CY7 (clone UCHT1), anti-CD158a/h (KIR2DL1/DS1)-FITC (clone 11PB6), and anti-human CD158e/k (KIR3DL1/DL2)-PE (clone 5.133) (Miltenyi Biotec, Bergisch Gladbach, Germany). Ki67 measurement was performed as follows: While vortexing, add 5 ml cold 75% ethanol dropwise into the cell pellet, following by incubation at -20°C for at least 2 hours. Subsequently, fixed cells were washed twice with 30-40 ml FACS staining buffer (PBS with 1% FBS) and then stained with Ki67 mAb-PE (clone SolA15, Rat/IgG2a eBioscience, San Diego, CA, USA). CD107a assay was performed as follows: NK cells cultured as described above for 5 days (Section 2.4, paragraphs 1 and 2) were cocultured with CFSE-labeled K562 cells in the presence of anti-CD107a-PE mAb and Cell Stimulation Cocktail (eBioscience, San Diego, CA, USA). After 1 h incubation, GolgiStop (BD Pharmingen, San Diego, CA) was added to the culture for an additional 4 h. Then, the samples were fixed and permeabilized using Cytofix/Cytoperm intracellular staining kits (BD Pharmingen, San Diego, CA) followed by staining with anti-IFN-*γ*-PE or anti-TNF-*α*-APC mAb (BD Pharmingen, San Diego, CA) and analyzed by FACS. For the FACS analysis of inflammatory cytokine production in NK cells, purified NK cells were incubated as described in cell proliferation analysis (Section 2.4, paragraph 1) for 72 h, subsequently stained with anti-IFN-*γ*-PE or anti-TNF-*α*-APC following by FACS analysis.

### 2.6. Quantitative RT-PCR and ELISA

RNAs were extracted from cell incubation as described above for 12 h or 24 h using TRIzol reagent (Invitrogen, Carlsbad, CA, USA). Reverse transcription (RT) was performed using RevertAid™ First Strand cDNA Synthesis Kit (Fermentas, Glen Burnie, MD, USA), followed by PCR assays using the primers as follows: human TNF-*α*, sense primer: 5′- CTC TTC TGC CTG CTG CAC TTT G -3′, antisense primer: 5′- ATG GGC TAC AGG CTT GTC ACT C -3′; human IFN-*γ*, sense primer: 5′- GAG TGT GGA GAC CAT CAA GGA AG -3′, antisense primer: 5′-TGC TTT GCG TTG GAC ATT CAA GTC -3′; human IL-10, sense primer: 5′- TCT CCG AGA TGC CTT CAG CAG A -3′, antisense primer: 5′- TCA GAC AAG GCT TGG CAA CCC A -3′; and GAPDH, sense primer: 5′- CTC CTC CTG TTC GAC AGT CAG C -3′, antisense primer: 5′- CCC AAT ACG ACC AAA TCC GTT -3′. Real-time PCR was performed in the Rotor-Gene 6000 real-time PCR detection system (Qiagen, Hilden, Germany). Reactions were completed in a 20 *μ*l volume containing a mixture of cDNA, specific primers of each gene, and the SYBR Green Master Mix (Takara, Otsu, Shiga, Japan). Gene expression was quantified relative to the expression of GAPDH and normalized to that measured in the control by standard 2^(-*ΔΔ*CT)^ calculation.

Culture supernatants were collected at 24 h after stimulation and cleared of debris by centrifugation. IFN-*γ*, TNF-*α*, and IL-10 expression levels in the culture supernatants were assayed with ELISA kits (eBioscience, San Diego, CA, USA) according to the manufacturer's instructions.

### 2.7. Western Blot Analysis

Cells incubated with IL-2 with/without 20 *μ*g/ml MBL for indicated time were harvested. The protein lysates were prepared using RIPA buffer (50 mM Tris, 150 mM NaCl, 1% NP-40, and pH 7.4), subsequently separated by SDS-PAGE and then transferred onto polyvinylidene difluoride (PVDF) membranes. Membranes were blocked in TBS containing 0.05% Tween-20 (TBST) and 5% BSA for 1 h at RT and incubated overnight at 4°C with the antibodies against STAT5 (clone 89/Stat5), phospho-STAT5 (clone 47/Stat5 (pY694)) (BD Pharmingen, San Diego, CA, USA), or GADPH (Sigma-Aldrich, St. Louis, MO, USA).

### 2.8. Statistical Analysis

All values were expressed as mean ± SEM. Unpaired Student's *t*-test or one-way ANOVA (for graphs including only 3 samples) was used as specified. *P* values < 0.05 were considered statistically significant.

## 3. Results

### 3.1. MBL Directly Binds to NK Cells

**Figure 1:**
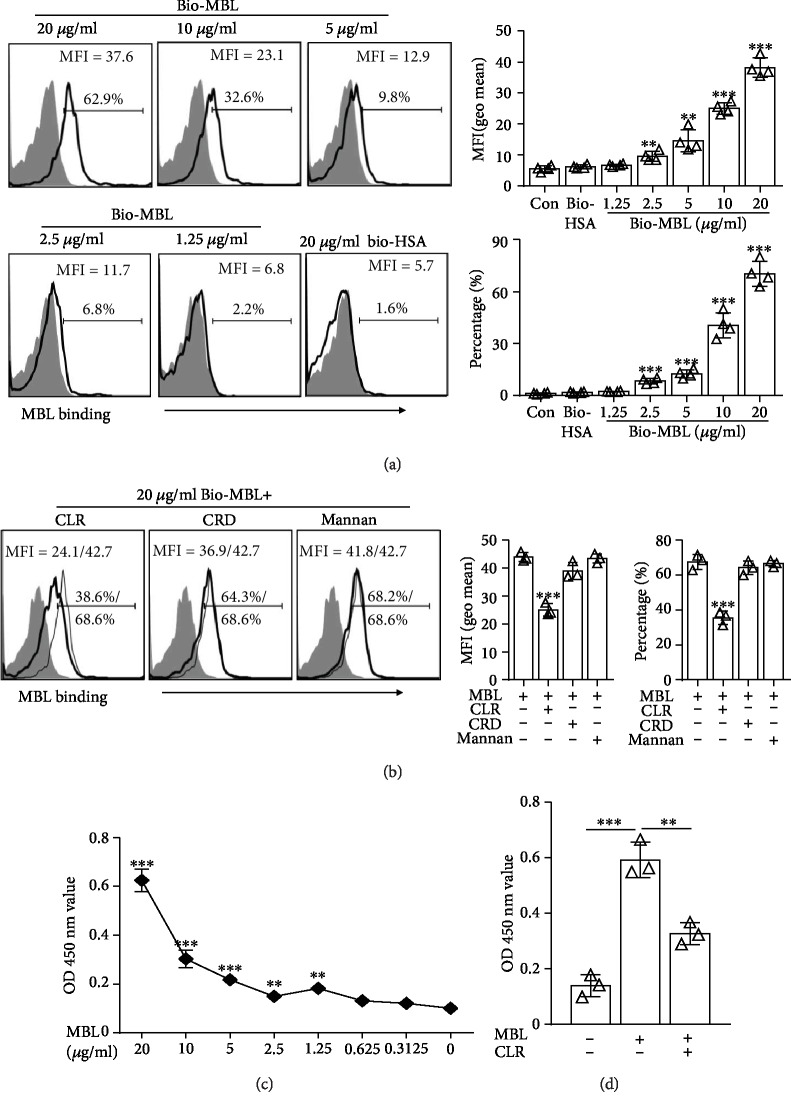
MBL directly bound to NK cells. (a) The binding of indicated concentration of bio-MBL or bio-HSA to purified NK cells was determined by FACS analysis (*n* = 4). (b) For competition assays, purified NK cells were preincubated with (thick lines) or without (thin lines) 50 *μ*g/ml mannan, CLR, or CRD of MBL following MBL binding analysis (*n* = 3). (c) MBL binding to NK cells was also determined using ELISA (*n* = 3). (d) 50 *μ*g/ml CLR of MBL pretreatment following MBL binding analysis was also used in ELISA to determine the binding domain of MBL. MFI = geometric mean. For FACS analysis, cells stained with streptavidin-FITC alone served as the control (shaded). Data are presented as means ± SEM (horizontal lines). ^∗^*P* < 0.05; ^∗∗^*P* < 0.01; ^∗∗∗^*P* < 0.001, compared to the control group without MBL in (a) and (c); compared to 20 *μ*g/ml bio-MBL alone in (b) and (d).

Our previous study revealed that human MBL could directly bind to kinds of immune cells such as monocytes and T cells [21, 22]. We hypothesized that MBL could also interact directly with NK cells. To confirm this hypothesis, we assessed the interaction between MBL and NK cells by FACS and cell-ELISA. As expected, MBL bound to purified NK cells in a dose-dependent manner (Figures 1(a) and 1(c)). Moreover, preincubation of NK cells with CLR of MBL before MBL binding significantly attenuated the MBL binding level but neither with CRD of MBL nor with mannan (Figures 1(b) and 1(d)). These results indicated that MBL could directly bind to NK cells partially via CLR of MBL.

### 3.2. MBL Regulates the Inflammatory Cytokine Production in NK Cells

**Figure 2:**
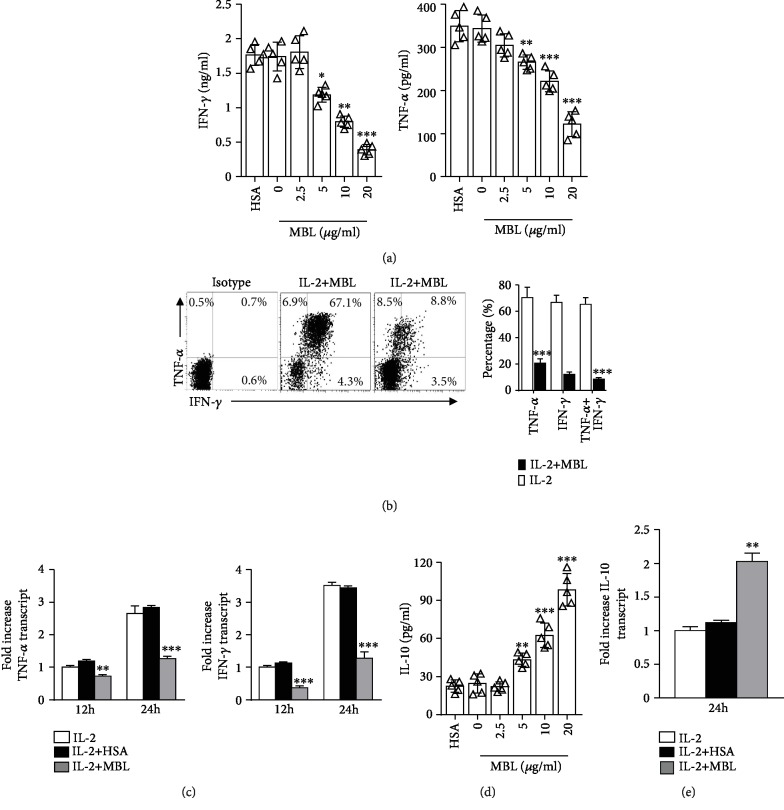
MBL regulated the inflammatory cytokine production in NK cells. Isolated human peripheral NK cells were stimulated with 100 U/ml recombinant IL-2 in the presence or absence of indicated concentration of MBL. Protein (a) and mRNA (c) levels of TNF-*α* and IFN-*γ* were analyzed by ELISA (*n* = 5, after 24 h incubation only) and qRT-PCR (*n* = 3) assays, respectively. (b) Representative FACS plots and statistical analysis are shown for IFN-*γ*/TNF-*α* production analysis after 72 h incubation (*n* = 3). Protein (d) and mRNA (e) levels of IL-10 were analyzed by ELISA (*n* = 5) and qRT-PCR (*n* = 3) assays after 24 h incubation, respectively. Data are presented as means ± SEM (horizontal lines). ^∗∗^*P* < 0.01; ^∗∗∗^*P* < 0.001, compared to the group treated with IL-2 alone.

We previously observed that MBL binding reduced the inflammatory response of monocyte and the T cell proliferation [16, 21]. Therefore, the observation that MBL binds to NK cells prompted us to investigate the effect of MBL on the NK cell activation. Initially, we assessed the inflammatory cytokine production of purified NK cells incubated in the presence of IL-2 with or without MBL. Notably, MBL attenuated production of the proinflammatory cytokines TNF-*α* and IFN-*γ* in NK cells upon stimulation compared with IL-2 alone as indicated by ELISA (Figure 2(a)), FACS (Figure 2(b)), and qRT-PCR (Figure 2(c)). However, the anti-inflammatory cytokine IL-10 was elevated in the presence of MBL upon stimulation (Figures 2(d) and 2(e)). Additionally, we observed that this regulation was dose-dependent (Figures 2(a) and 2(d)). These data suggest that MBL binding could regulate the inflammatory cytokine production in NK cells during priming by IL-2.

### 3.3. MBL Reduces NK Cell Proliferation

**Figure 3:**
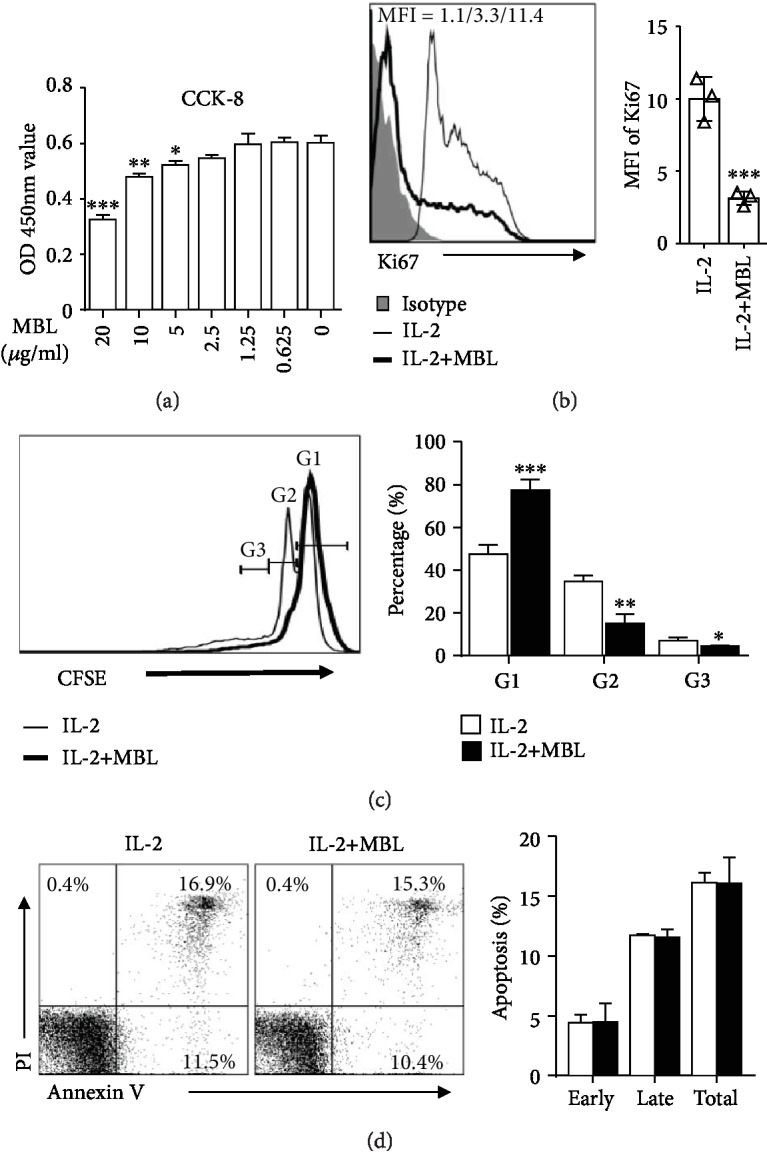
MBL treatment reduced NK cell proliferation. CFSE-labeled or unmarked purified NK cells were stimulated with 100 U/ml recombinant IL-2 in the presence or absence of MBL for 5 days. (a) Effects of different concentrations of MBL on the expansion of NK cells were assessed by the CCK-8 assay. (b) For Ki67 measurement, cells were fixed and stained by anti-Ki67, followed by FACS analysis. (c) Representative division histogram (left panel) and the percentages of CFSE-labeled NK cells in each peak of division (G, generation) (right panel) were calculated by FACS analysis. (d) The cell apoptosis was analyzed by FACS. The percentages represent the frequency of the cells undergoing early apoptosis (Annexin V^+^7-AAD^−^) or late apoptosis (Annexin V^+^7-AAD^+^). Data are presented as means ± SEM (horizontal lines) (*n* = 3). ^∗^*P* < 0.05; ^∗∗^*P* < 0.01; ^∗∗∗^*P* < 0.001, compared to the group without MBL protein.

On the basis of the observation that MBL regulates NK cell inflammatory cytokine production upon stimulation, we speculated that MBL could also influence NK cell proliferation. Therefore, we assess the proliferation level of NK cell cultured with or without MBL upon IL-2 stimulation. To determine the MBL effect on purified NK cell proliferation, NK cells were cultured for 5 days in the presence of IL-2 with MBL or HSA. We observed that the NK cell proliferation decreased after MBL treatment compared to the control by the analysis of CCK-8 (Figure 3(a)), anti-Ki67 (Figure 3(b)), and CFSE staining (Figure 3(c)) by FACS. In addition, we excluded the potential that MBL may reduce NK cell proliferation through apoptosis induction (Figure 3(d)). Thus, these results revealed that MBL could reduce NK cell proliferation upon IL-2 stimulation.

### 3.4. MBL Attenuates Expression of NK Cell Activation Markers

**Figure 4:**
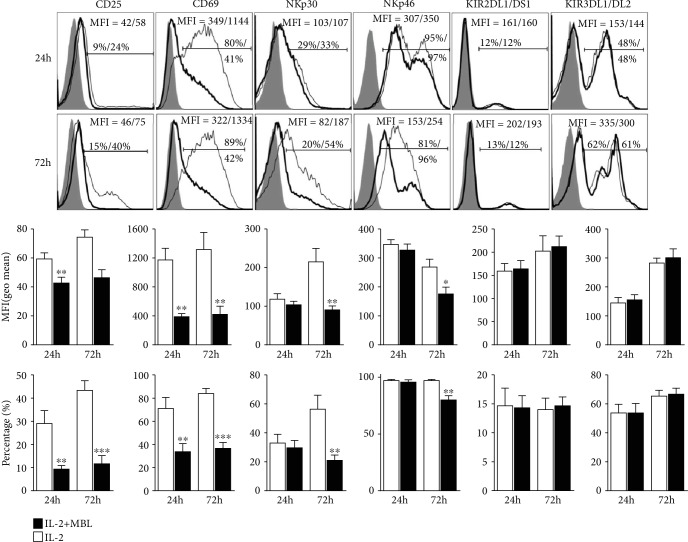
MBL attenuated expression of NK cell activation markers. NK cells were stimulated with 100 U/ml recombinant IL-2 in the presence or absence of 20 *μ*g/ml MBL for 24 hours or 72 h. Representative histogram and statistical analysis showed the expression profiles of indicated receptors in NK cells treated with (thick lines) or without MBL (thin lines). The shaded curves describe cells stained with corresponding isotype control. MFI = geometric mean. ^∗^*P* < 0.05; ^∗∗^*P* < 0.01; ^∗∗∗^*P* < 0.001, compared to the group with IL-2 alone. Data are presented as means ± SEM (horizontal lines) (*n* = 3).

To characterize the activation status of NK cells treated with MBL, we determined the expression level of surface NK cell receptors by FACS. Notably, CD69 and CD25, which are the activation markers of NK cells, decreased when treated with MBL compared to the control (Figure 4). Additionally, MBL treatment notably decreased the expression of activation receptor NKp30 and NKp46, whereas the killer cell immunoglobulin-like receptors (KIR) KIR2DL1/DS1 and KIR3DL1/DL2 did not change significantly (Figure 4). According to these results, we found that MBL impaired NK cell activation status upon stimulation.

### 3.5. MBL Causes Defects of NK Cell Cytotoxic Function

**Figure 5:**
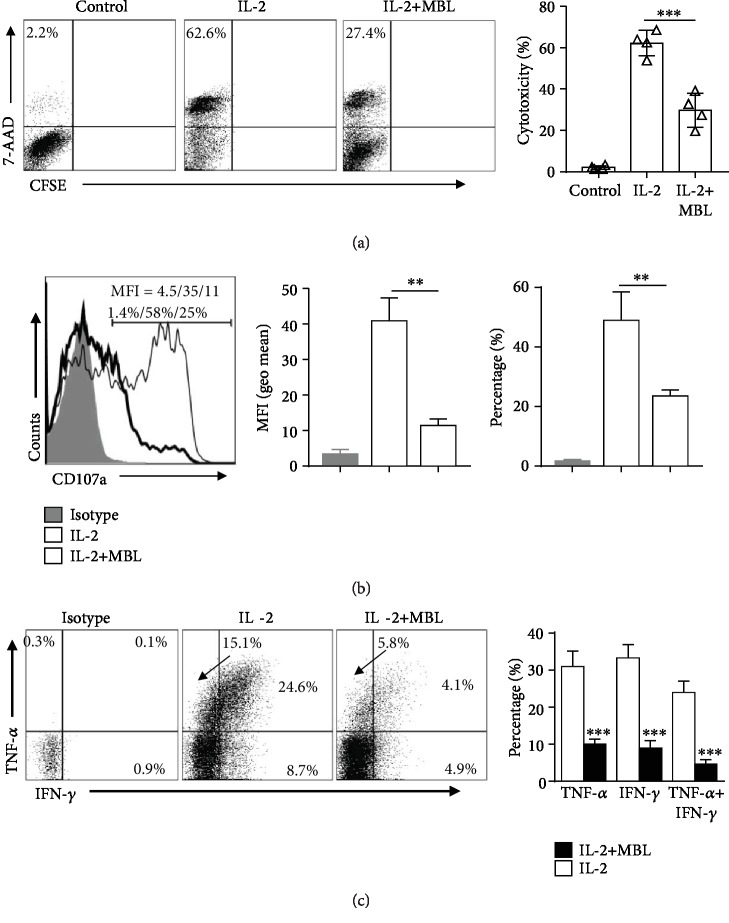
MBL impaired cytotoxicity of NK cells. The cytotoxicity of NK cells (LAK activity) toward K562 cells (a), expression of CD107a (b), and TNF-*α*/IFN-*γ* (c) were evaluated by FACS after coculture of stimulated NK cells and K562 cells with the additional 4 hours, K562 cells alone as the control; CFSE-labeled NK cells were stimulated with 100 U/ml recombinant IL-2 in the presence or absence of 20 *μ*g/ml MBL for 5 days. The percentages represent the frequency of CD107a^+^ NK cells, and the numerical values represent the MFI of CD107a in NK cells (b). MFI = geometric mean. ^∗∗^*P* < 0.01; ^∗∗∗^*P* < 0.001, compared to the group with IL-2 alone. Data are presented as means ± SEM (horizontal lines) (*n* = 3).

We next examined whether impaired NK activating status by MBL could lead to functional defects of NK cells. Therefore, the lymphokine-activated killing (LAK) activity of NK cells stimulated by IL-2 in the presence or absence of MBL was monitored. As seen in Figure 5(a), MBL-treated NK cells showed reduced cytotoxicity against its natural targets (K562), compared with the activated NK cells with IL-2 alone. In addition, the reduction in the cytotoxicity of MBL-treated NK cells was correlated directly with a substantial decrease in CD107a degranulation (Figure 5(b)). These data demonstrate that MBL causes functional defects in cytolysis in NK cells and further suggest that MBL regulate NK cell activation. Notably, in coculture with K562 cells, MBL-treated NK cells produced fewer IFN-*γ* and TNF-*α* compared with NK cells treated with IL-2 alone (Figure 5(c)). Thus, these results indicated that MBL treatment impaired the cytotoxicity of NK cell upon stimulation.

### 3.6. Activation of STAT5 Was Impaired in MBL-Treated NK Cells during IL-2 Stimulation

**Figure 6:**
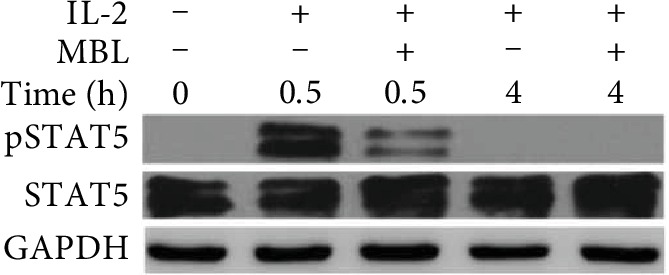
MBL inhibited IL-2-induced activation of STAT5 in NK cells. Purified human peripheral NK cells were stimulated with recombinant 100 U/ml human IL-2 in the presence or absence of 20 *μ*g/ml MBL. Cell lysate was harvested at the indicated time point and then analyzed using immunoblotting for phosphorylation of STAT5 and total STAT5. GAPDH served as a loading control. The representative image of at least three independent experiments with similar results was shown.

As IL-2 was shown previously to stimulate STAT signaling pathways, especially STAT5 [24], we measured the effect of MBL on the activation of STAT5. Upon IL-2 stimulation, STAT5 phosphorylation was induced in NK cells; however, it appeared to reduce significantly in MBL-treated activated NK cells. Phosphorylation of STAT5 reduced without affecting the total amount of STAT5 protein (Figure 6). Taken together, these data suggest that MBL interaction leading to decreased STAT5 phosphorylation might underlie the functional defects in the proliferation, cytolysis, and cytokine production by MBL.

## 4. Discussion

MBL, which belongs to the collectin family of C-type lectins, can recognize the carbohydrate motif and then activate the lectin complement pathway, enhance phagocytosis, and modulate inflammation [25]. In the current study, we have discovered that the interaction of plasma MBL with human peripheral NK cells markedly suppressed the inflammatory cytokine production and activation status in NK cells upon IL-2 stimulation. In addition, MBL inhibited the proliferation and LAK of these IL-2-activated NK cells, accompanied by decreased STAT5 phosphorylation. This finding, to our knowledge, is the first report to show that MBL engagement modulates NK cell activity, which may provide new insight into the regulatory mechanism of MBL in inflammation and the immune response.

The execution of the immunoregulatory function of the collectin family apart from complement activation was first studied in the surfactant proteins [26]. Several studies demonstrated that surfactant protein- (SP-) A and SP-D, which are highly homologous to MBL, regulated the functions of various immune cells and modulated the immune response [27–31]. Additionally, it has been reported that ficolin-A, a lectin analogous to MBL, could also exert their immunomodulatory function [32, 33]. Although we and others found that MBL played as pleiotropic immune modulator affecting numerous cell types of innate and adaptive immunity [16, 19–21, 32, 34], the underlying mechanisms have not been fully elucidated yet.

Besides their potent cytolytic activity, NK cells also play a crucial immunomodulatory role in inflammation and the immune response via producing cytokines as well as interacting with other immune and nonimmune cells [35]. Emerging evidence indicated that resting NK cell functional priming is stringently controlled by the integration of various immune modulators [10]. Recent studies demonstrated that SP-D could bind to NK cells and modulate their function [36, 37]. Additionally, a previous study found that ficolin-1 could attach to the NK cell surface via the pathogen recognition domain of ficolin-1, although whether this interaction might affect NK cell function was still unclear [38]. In this study, we observed that MBL could interact directly with NK cells and efficiently attenuate NK cell activation, thereby extending our understanding of the immunomodulatory role of collectins in NK cell activity.

An early study showed that MBL could conjugate to several autologous immune cell types including CD56^+^ PBMC, and the binding was optimal at supraphysiological MBL concentrations [39]. Indeed, our present results found that human plasma MBL could bind directly with purified peripheral NK cells in a concentration-dependent manner. Although a previous study showed that MBL could bind to differently glycosylated ligands on the surface of B cells [39], our current work suggests that the interaction of MBL with NK cells was partially via CLR of MBL. These results were consistent with our recent study which observed that MBL could conjugate to calreticulin (CRT) on the surface of activated T cells via CLR of MBL [21]. Additionally, it is also supported by the previous studies that reported NK cells expressed the MBL receptors CRT [40] and C1qRp [41]. Therefore, our present finding confirmed the direct interaction of MBL with NK cells and pointed toward the potential immunoregulatory effects of MBL on NK cells. Future studies will investigate how the interaction of NK cell-MBL occurs and then influences the intercellular signaling pathways.

It is well known that IL-2, one of the first cytokines discovered, plays a fundamental role in the activation and expansion of NK cells [42]. Several studies have shown that IL-2 is necessary for the adaptive-innate lymphocyte cross-talk that tunes NK cell reactivity in the immune network [43]. Upon stimulation with IL-2, NK cells were activated and produce several inflammatory cytokines, alter the expression of the surface markers, and develop a robust cytotoxic activity [44]. Of note, proinflammatory cytokines TNF-*α* and IFN-*γ* are members of the most potent effector cytokines secreted by NK cells upon activation [45]. Additionally, NK cell-derived IFN-*γ* could trigger the differentiation of proinflammatory macrophages and promote inflammation [46]. Furthermore, it has been reported that the production of anti-inflammatory cytokine IL-10 in regulatory NK cells could negatively regulate the immune response [47]. In this study, we demonstrated that MBL treatment markedly reduced the IL-2-induced production of TNF-*α* and IFN-*γ* in NK cells, whereas the IL-10 level was elevated. These results were consistent with our previous finding that exogenous MBL reduces the inflammatory cytokine production of monocytes *in vitro* [16]. Additionally, we and others found that MBL deficiency elevated the inflammatory cytokines production both in mice [19] and humans [18]. Furthermore, this finding could be explained by an early study suggesting that the binding of MBL to autologous cells is most likely to take place at inflammatory loci, where it recognizes endogenous ligands and induces the release of anti-inflammatory mediators [48]. Collectively, our present study provided evidence that MBL might inhibit the inflammatory response via the impairment of NK cell activity.

Given that NK cells could proliferate upon activation [49], it is not surprising that IL-2 promoted the expansion of NK cells whereas MBL treatment could attenuate this expansion. Of note, NK cells could also alter the expression of cell surface markers upon stimulation. Emerging evidence showed that CD69 and CD25 expression was upregulated following NK cell activation, which was the marker of activated NK cells [50]. Additionally, NK cell priming usually depended upon the integration of signals derived from the activating and inhibitory receptors expressed on the NK cell surface [51]. Our present study found that MBL involvement decreased the CD69 and CD25 expression, as well as the activating receptors NKp46 and NKp30. However, the KIR KIR2DL1/DS1 and KIR3DL1/DL2 did not change. These findings further confirmed that MBL involvement could attenuate NK cell priming and suggested this effect might affect NK cell priming depending on the expression of activating receptors.

In fact, activated NK cells could recognize and defend against target cells by increasing the number and size of cytoplasmic granules [10]. It is well known that IL-2 treatment could notably augment their cytotoxic function (conventionally referred to as the LAK activity, even upon weak stimulation [50]). As expected, MBL treatment significantly impaired IL-2-induced LAK activity of NK cells. This impairment was indicated by the reduced cytotoxicity of MBL-treated NK cells against K562 cells and a substantial decrease in CD107a degranulation, as well as the reduction of IFN-*γ*- and TNF-*α*-expressing NK cell populations. Therefore, this finding indicated that MBL causes functional defects in cytolysis of NK cells and further confirmed that MBL regulated NK cell activity.

It has been reported that IL-2 mediated its effects mainly through the Janus tyrosine kinase (JAK)/signal transducer and activator (STAT) pathway [52]. Upon activation, STAT1, STAT3, and STAT5 translocate to the nucleus and activate target genes, contributing to the generation of LAK activity and IFN-*γ* expression and increased surface expression of CD69 on NK cells [44]. Additionally, Stat5b is essential for NK cell proliferation and cytolytic activity [53]. As expected, our present results observed that IL-2 did induce the STAT5 phosphorylation whereas MBL treatment caused the notable reduction of STAT5 phosphorylation. These data indicated that the MBL interaction with NK cells might regulate their activity via affecting the intercellular signaling pathways.

## 5. Conclusions

In summary, we discovered that MBL serves as a regulator of NK cell activation by its conjugation with NK cells, suggesting that MBL might execute its anti-inflammatory function in the inflammatory loci via direct interaction with NK cells. Future work is necessary to further define the underlying mechanisms of MBL-mediated suppression of NK cell activity in NK cell-mediated inflammation and immune response *in vivo*, providing new opportunities for therapeutic intervention in NK cell-associated inflammatory and autoimmune diseases.

## Data Availability

The data used to support the findings of this study are included within the article.

## Abbreviations

NK cells: Natural killer cells
IL-2: Inerleukin-2
CRD: Carbohydrate recognition domain
CLR: Collagen-like region
IFN: Interferon
FACS: Flow cytometry
HSA: Human serum albumin
ILC: Innate lymphoid cells
LAK: Lymphokine-activated killing
MBL: Mannan-binding lectin
PBMC: Peripheral blood mononuclear cell
STAT5: Signal transducers and activators of transcription 5
TNF-*α*: Tumor necrosis factor-alpha.
